# Exploring disease-causing traits for drug repurposing in critically ill COVID-19 patients: A causal inference approach

**DOI:** 10.1016/j.isci.2023.108185

**Published:** 2023-10-12

**Authors:** Hannes A. Baukmann, Justin L. Cope, Colin Bannard, Alexander R.E.C. Schwinges, Margaretha R.J. Lamparter, Sarah Groves, Charles N.J. Ravarani, Borko Amulic, Joern E. Klinger, Marco F. Schmidt

**Affiliations:** 1 biotx.ai GmbH, Am Mühlenberg 11, 14476 Potsdam, Germany; 2 University of Manchester, Oxford Road, Manchester M13 9PL, UK; 3 School of Cellular and Molecular Medicine, Faculty of Life Sciences, University of Bristol, Bristol BS8 1DT, UK

**Keywords:** Genetics, Immunology, Microbiology, Association analysis

## Abstract

Despite recent development of vaccines to prevent SARS-CoV-2 infection, treatment of critically ill COVID-19 patients remains an important goal. In principle, genome-wide association studies (GWASs) provide a shortcut to the clinical evidence needed to repurpose existing drugs; however, genes identified frequently lack a causal disease link. We report an alternative method for finding drug repurposing targets, focusing on disease-causing traits beyond immediate disease genetics. Sixty blood cell types and biochemistries, and body mass index, were screened on a cohort of critically ill COVID-19 cases and controls that exhibited mild symptoms after infection, yielding high neutrophil cell count as a possible causal trait for critical illness. Our methodology identified CDK6 and janus kinase (JAK) inhibitors as treatment targets that were validated in an *ex vivo* neutrophil extracellular trap (NET) formation assay. Our methodology demonstrates the increased power for drug target identification by leveraging large disease-causing trait datasets.

## Highlights

• Neutrophil cell count plays a causal role for severe COVID-19 disease progression
• Validated CDK6 and JAK inhibitors as treatment targets for severe COVID-19
• Large disease-causing trait datasets increase the power of drug target identification

## Graphical abstract

**Figure:**
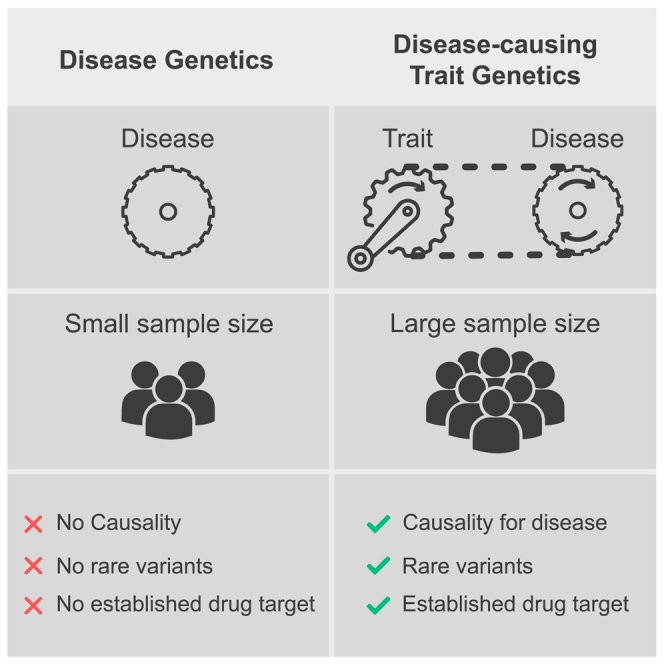

## Introduction

The phenotype of critically ill coronavirus disease 2019 (COVID-19) status substantially differs from mild or moderate disease, even among hospitalized cases, by an uncontrolled overreaction of the host’s immune system^1^^,^^2^^,^^3^—a so-called virus-induced immunopathology^4^—resulting in acute respiratory distress syndrome (ARDS). The molecular mechanism leading to critical illness due to COVID-19 is still unclear. Identifying causal risk factors is central for prevention and treatment. Nonetheless, there is evidence that susceptibility and overreaction of the immune system to respiratory infections are both strongly heritable.^5^^,^^6^ A series of genome-wide association (GWA) studies (GWASs) have been conducted to investigate disease pathogenesis in order to find mechanistic targets for therapeutic development or drug repurposing.^7^^,^^8^^,^^9^^,^^10^ Treating the disease remains a top priority despite the recent development of vaccines preventing severe acute respiratory syndrome coronavirus 2 (SARS-CoV-2) infection due to the threat of new vaccine-resistant variants.

The results of 46 GWA studies comprising 46,562 COVID-19 patients from 19 countries have been combined in three meta-analyses by the COVID-19 Host Genetics Initiative.^10^ Their study identified 15 independent genome-wide significant loci associations for COVID-19 infection in general, of which six were found to be associated with critical illness due to COVID-19: 3p21.31 close to *CXCR6*, which plays a role in chemokine signaling, and *LZTFL1*, which has been implicated in lung cancer; 12q24.13 in a gene cluster that encodes antiviral restriction enzyme activators; 17q21.31 containing the *KANSL1* gene, which has been previously reported for reduced lung function; 19p13.3 within the gene that encodes dipeptidylpeptidase 9 (*DPP9*); 19p13.2 encoding tyrosine kinase 2 (*TYK2*); and 21q22.11 encoding the interferon receptor gene *IFNAR2*. The functions of the genes associated with these six loci are either related to host antiviral defense mechanisms or mediators of inflammatory organ damage.

Nonetheless, using GWAS data for drug development has several general drawbacks, which are particularly evident in the case of COVID-19. First, none of the reported genes encodes an established drug target, limiting the actionability of the discoveries. Rather, the exact function of the gene variants found in patients with critical illness due to COVID-19 is unclear. Therefore, it is questionable whether manipulation of the function of the gene product by a drug is an effective approach. Second, GWA studies only offer correlations of genes with diseases, not causal relationships, which are important for drug development. Third, due to the currently limited sample size of GWA study datasets (<5,000 individuals), biologically relevant rare variants with small effect sizes cannot be detected.

**Figure 1:**
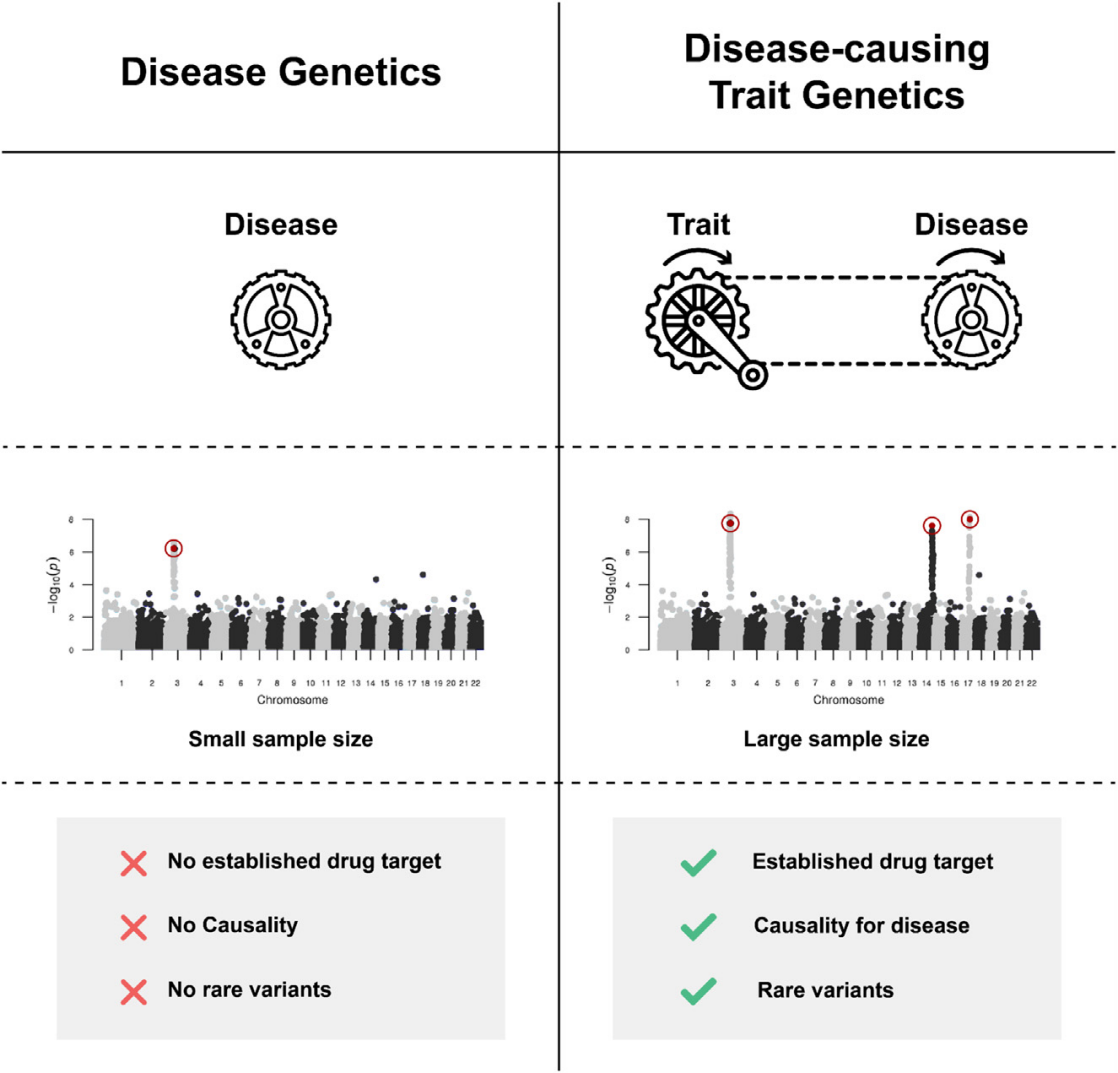
Disease genetics vs. disease-causing trait genetics for the identification of drug targets Instead of focusing on disease genetics, genetics of disease-causing traits has three advantages. First, disease-causing traits are often more likely to be associated with known druggable targets such as enzymes or receptors. Second, unlike a disease-associated gene, the function and, from there, causality of a gene for a trait is easier to verify. Third, the sample size of trait datasets is far greater than that of datasets containing cases and controls specifically for COVID-19.

**Figure 2:**
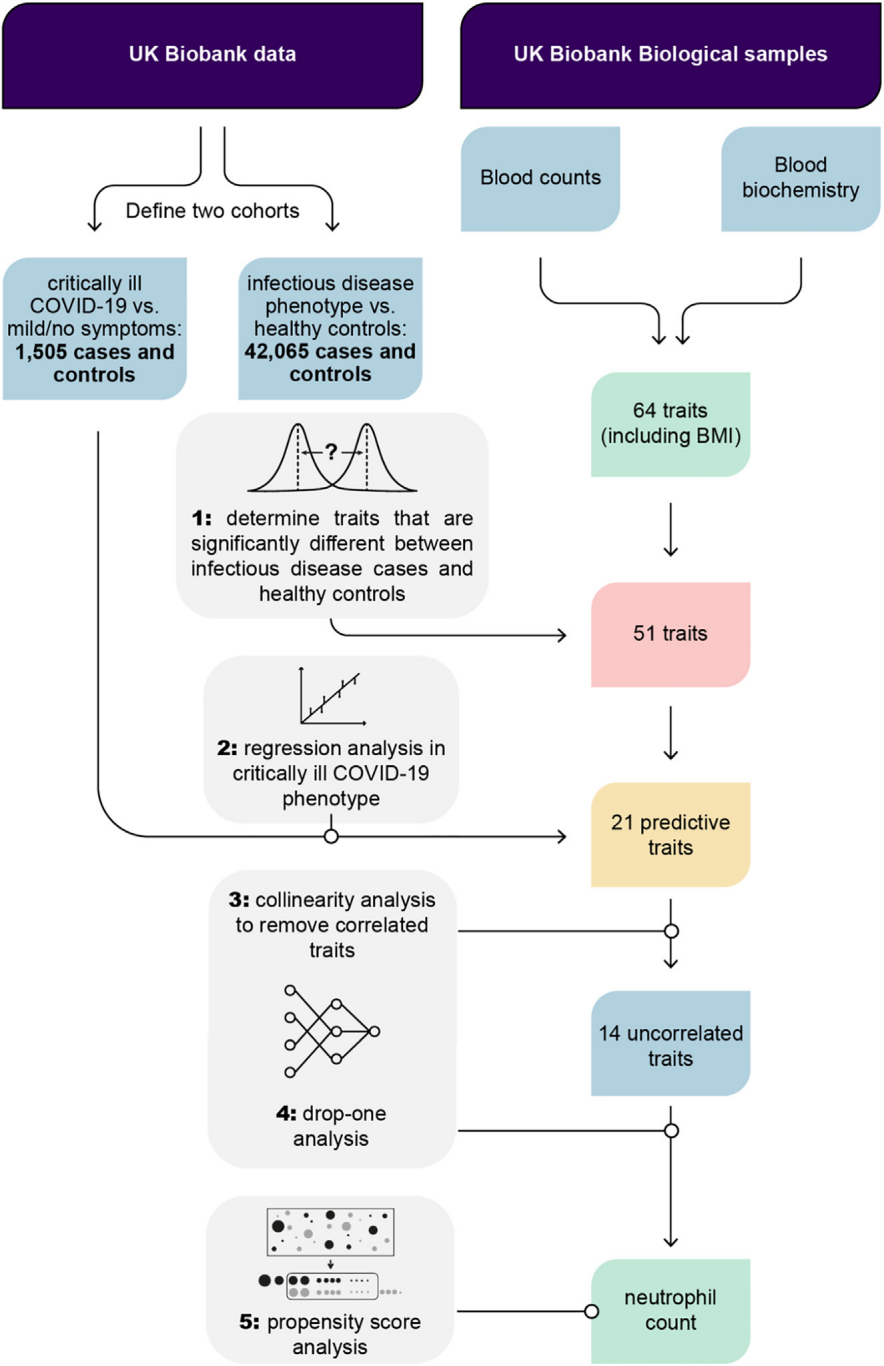
The causal inference analysis workflow to identify traits leading to critical illness due to COVID-19 We identified significant differences in 64 candidate predictive traits between an infectious disease cohort and healthy controls. We used regression models to investigate the effect of these traits on critically ill COVID-19 cases compared to asymptomatic controls. Because highly dependent traits would not be significant in drop-one analysis, we first used collinearity testing to remove correlated traits. Using drop-one analysis, we identified neutrophil cell count as a trait that has a unique effect on critical illness in COVID-19. Finally, we showed that neutrophil count is causal for severe COVID-19 using propensity score analysis.

Here, we present an approach overcoming the aforementioned shortcomings for drug development or repurposing, which is based on the genetics of disease-causing traits rather than the genetics of disease (Figure 1). Using data from the UK Biobank,^11^ critically ill COVID-19 patients of European ancestry were matched with a control group of COVID-19 patients with mild illness (see STAR methods). Traits that differed significantly in cases and controls were further examined for causality with respect to critical illness in COVID-19 (Figure 2). The genetics of these traits were further investigated to identify and test established target genes for drug repurposing to establish their precise mechanism within the specific disease context. Focusing on the genetics of disease-causing traits reveals three advantages. First, disease-causing traits can more likely be manipulated with a drug via largely known druggable targets such as enzymes or receptors. Second, unlike a disease-associated gene, the function and, from there, possible causality of a gene for a trait are more robustly inferred. Third, the sample size of trait datasets is far greater than that of datasets specifically for COVID-19. For example, datasets on traits such as blood biochemistry often include >500,000 cases. Therefore, biologically relevant rare variants with small effect sizes can be detected.

## Results

### Screening for traits associated with infectious disease

Using UK Biobank data,^11^ we identified 42,065 individuals with respiratory infections, ARDS, influenza, and pneumonia, which serve as our infectious disease cohort (see STAR methods). In order to explore how the infectious disease cohort differs from healthy controls, we screened 64 candidate traits (33 blood cell counts and 30 blood biochemistry markers, and body mass index [BMI]) that might be predictive for disease. Importantly, the traits were measured years before the individuals were affected by the disease, i.e., when they joined the biobank and not after they were infected with COVID-19. We observed Bonferroni-corrected statistically significant differences (*p* < α/n = 0.05/64)^12^ in 51 traits confirmed by independent two-sample *t*-test and two-sided Wilcox rank-sum test (Figures 2 and S1).

### Regression modeling

Furthermore, we identified 1,505 patients who were hospitalized due to SARS-CoV-2 infection and who required respiratory support and/or died due to infection.^13^ These patients were defined as cases and matched to controls that were infected with SARS-CoV-2 but showed no or only mild symptoms (see STAR methods). Carrying over the 51 traits identified in the previous step, we used regression modeling to investigate the effect of these traits on critically ill COVID-19 status. Out of the 51 traits, 21 traits were significant predictors of critical illness due to COVID-19 with a Bonferroni-corrected significance threshold of *p* < α/n = 0.05/51 (Figure 2 and Table S1).

### Collinearity analysis

Collinearity is the correlation between predictor variables in a regression model. Therefore, collinearity between traits would impact the results of the drop-one analysis. We first identified traits to remove in order to solve this issue. Seven traits were thus excluded from further analysis: leukocyte count, reticulocyte count, reticulocyte percentage, high light scatter reticulocyte percentage, immature reticulocyte fraction, HDL cholesterol, and glycated hemoglobin (HbA1c) (Figures 2, S2, and Table S2).

### Drop-one analysis

Drop-one analysis compares all possible models that can be constructed by dropping a single model term and evaluating its impact on the regression model. The analysis revealed that only neutrophil count explains unique variance in critically ill COVID-19 status to a Bonferroni-corrected significance threshold of *p* < α/n = 0.05/14 (Figure 2 and Table S3).

### Propensity score analysis

Propensity score analysis is a technique for estimating the effect of a treatment on an outcome independent of covariates. We employed propensity score stratification using the propensity function of Imai and van Dyk^14^ in order to estimate the effect of the treatment on critical illness in COVID-19 independent of the known risk factors for critical illness in COVID-19: age, sex, BMI, C-reactive protein (as a proxy for autoimmune disease), cystatin C (as a proxy for cardiovascular disease), alanine aminotransferase (as a proxy for chronic liver disease), and creatinine (as a proxy for chronic kidney disease). Neutrophil count was found to have a significant effect on critical illness in COVID-19 (p = 1.8228E-06, estimated effect = 0.13177 ± 0.028456) (Figure 2 and Table S4).

### Trait genetics analysis

We next focused on the genetics of neutrophil cell count by performing a GWA analysis using the entire UK Biobank (471,532 participants, of which 444,109 had measurements available; Figures S3 and S4). We compared our results with previously published statistics from the NHGRI-EBI GWAS catalog^15^ and were able to confirm them. Subsequently, we collected genes and approved drugs for variants exceeding a significance threshold of -log p = 80 (Table S5). Since no clear drug-to-gene assignment was possible for the gene variants of the human leukocyte antigen (HLA) haplotype on chromosome 6 due to its genetic complexity and high level of diversity, we focused on other significant gene variants. The most significant gene variants were found in the *PSMD3* gene which is targeted by bortezomib and carfilzomib. This is followed by gene variants in the *CDK6* gene, which is associated with the drugs palbociclib, ribociclib, fulvestrant, abemaciclib, trilaciclib, apremilast, and dexamethasone. Furthermore, gene variants and associated drugs were found in the genes *NR1D1* (lithium), *THRA* (levothyroxine, liothyronine, aspirin, and lithium), *CXCR2* (clotrimazole, acetylcysteine, and ibuprofen), *PLAUR* (filgrastim and ruxolitinib), and *JAK1* (baricitinib and ruxolitinib).

Bortezomib and carfilzomib are proteasome inhibitors approved for cancer therapy, whereas *PSMD3* encodes one of the non-ATPase subunits of the 19S regulatory lid.^16^ Therefore, bortezomib and carfilzomib do not bind directly to the protein encoded by the *PSMD3* gene. In contrast, the drugs already approved for breast cancer abemaciclib, ribociclib, trilaciclib, and palbociclib bind directly to the protein encoded by the *CDK6* gene, cyclin-dependent kinase 6 (CDK6). The same was true for the janus kinase (JAK) inhibitors, which are used for rheumatoid arthritis (baricitinib) as well as myelofibrosis and polycythemia vera (ruxolitinib).

We also conducted GWA analyses with the cases and controls defined earlier and a random population of the same size (n = 3,010) (Figures S5 and S6, respectively). In both cases, we could not identify variants with genome-wide significance. Statistical power analysis shows that this is due to the lack of statistical power in GWA analyses given the sample examined (Figure S7).

### Neutrophil extracellular trap (NET) formation inhibition

**Figure 3:**
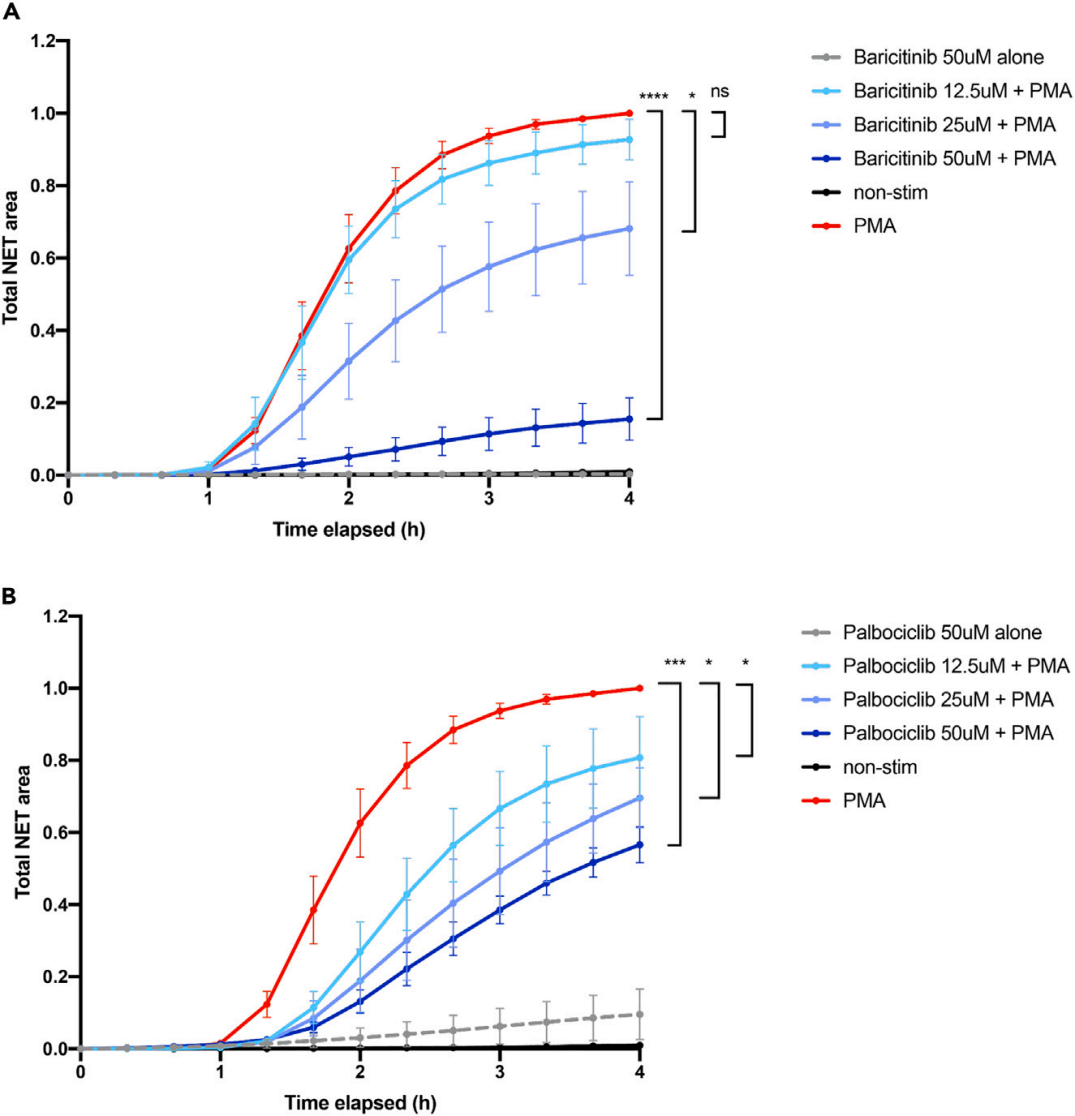
Baricitinib and palbociclib inhibit NET formation (A and B) Neutrophils from healthy donors (n = 6) were incubated with baricitinib (A) or palbociclib (B) for 45 min at the indicated concentrations, before stimulation of NETosis with PMA (50 nM). Total NET area was quantified using live-cell imaging over 4 h in the presence of SYTOX green. Statistical significance at endpoint (4 h) was determined using the Wilcoxon signed-rank test. ∗<0.05; ∗∗∗<0.001.

Excessive neutrophil activity has been proposed to cause systemic and localized inflammatory damage in severe COVID-19.^17^ Specifically, dysregulated release of chromatin in the form of NETs has been linked with poor prognosis and lung failure.^18^ We therefore tested the drugs identified in the earlier analysis (as well as colchicine, another proposed drug for severe COVID-19) for their ability to modulate NET production *ex vivo*. To determine the appropriate concentrations of carfilozimib, palbociclib, baricitinib, colchicine, and sabizabulin, we first incubated primary human neutrophils with various dilutions of the compounds and measured toxicity. We found that all four drugs were well tolerated up to 50 μM (Figure 3). Next we stimulated NETosis with the strong inducer phorbol myristate acetate (PMA), in either the presence of the compounds (up to maximum concentration of 50 μM) or vehicle control (DMSO). Colchicine, sabizabulin, and carfilizomib did not affect NET formation (Figure S8), while palbociclib had a partial inhibitory effect (Figure 3), as previously reported.^19^ The strongest inhibition of NET formation was observed with baricitinib (Figure 3), which reduced NETosis by approximately 80% (Figure 3, n = 6 blood donors).

NET release occurs via two known pathways, both of which require production of reactive oxygen species (ROS) but differ in the source of oxygen radicals. PMA induces NETs via the NADPH Oxidase (NOX2)-dependent pathway,^20^ while calcium ionophores trigger NOX2-independent NETs. We tested whether the drugs (colchicine, sabizabulin, carfilzomib, palbociclib, and baricitinib) can also affect the NOX2-independent pathway, by quantifying their effect on NETs triggered by the calcium ionophore A23187, in three healthy donors. Baricitinib suppressed NOX2-independent NETs, although this was not statistically significant due to small sample size, while palbociclib unexpectedly led to increased NETosis in this pathway (Figure S9). In summary, baricitinib demonstrates superior NET inhibition capacity, potentially explaining its success in clinical trials for critical illness in COVID-19.^21^

### Mendelian randomization

Mendelian randomization (MR; see STAR methods) is a robust and accessible tool to examine the causal relationship between an exposure variable and an outcome from GWAS summary statistics.^22^ We employed two-sample summary data MR to further validate causal effects of neutrophil cell count variants on critical illness due to COVID-19. We used independent GWAS summary data for neutrophil cell count (exposure) published by Vuckovic et al.^23^ and summary data for critical illness in COVID-19 (outcome) published by the COVID-19 Host Genetics Initiative.^10^ The inverse variance weighted (IVW) result was significant with a p value of 0.01199 when a lenient clumping parameter of r = 0.2 was used yielding 1,581 single nucleotide polymorphisms (SNPs), whereas we observed no significant IVW result when we used strict clumping parameters of r = 0.01 and 567 SNPs (Table S6).

## Discussion

Computational methods to drug repurposing are a very active field of development with different approaches often complementing each other (e.g., RNA sequencing [RNA-seq], causal inference, polygenic scoring, etc.).^24^^,^^25^ Solely relying on drug targets identified in classical GWA studies rarely succeeds. That is because GWA hits correlate with disease but provide no indication of the causal pathway to disease. Moreover, rare variants with small effect sizes are not found because of sample sizes that are drastically limited by the number of patients available for a study. Here, we describe a method that prioritizes the identification of traits with a causal role in disease pathogenesis. Subsequent investigation of the genetics of the disease-causing traits enables the discovery of drug targets that would not be found in classical GWA studies because of typically small sample sizes.

Our approach was as follows. First, we identified significant differences in 64 predictive characteristics between a cohort of infectious disease and healthy control subjects from the UK Biobank. Using regression models, we examined the effects of these characteristics on severely ill COVID-19 cases compared with mild control cases. Because highly correlated characteristics would be missed in a drop-one analysis, collinear (non-independent) characteristics were first removed. Of the remaining characteristics, neutrophil count was identified as having a unique association with critical illness in COVID-19 independent of other characteristics. Age, male gender, obesity, type 2 diabetes, cardiovascular disease, and chronic liver and kidney disease have been previously described as risk factors for the severe course of COVID-19.^26^ Based on the characteristics measured in the UK Biobank, we used these risk factors or surrogate factors as confounders in the propensity score analysis. Finally, the propensity score analysis provides evidence for the causal effect of neutrophil count on severe COVID-19 independent of these risk factors.

Neutrophil cell count is a general marker for infectious/autoimmune diseases,^19^ and selecting a drug based on modulating its levels does not provide a COVID-19-specific treatment. The specific context that a disease creates is important; for example, lupus and COVID-19 are both fueled by neutrophil activation but, in the latter, the neutrophils exert their destructive effects in the lung tissue. It has thus not been our aim to identify drug targets with the best disease specificity, but to identify the mechanism that has a specific impact on the disease. It is possible that the drug targets identified could be further repurposed for other diseases on top of COVID-19.

**Figure 4:**
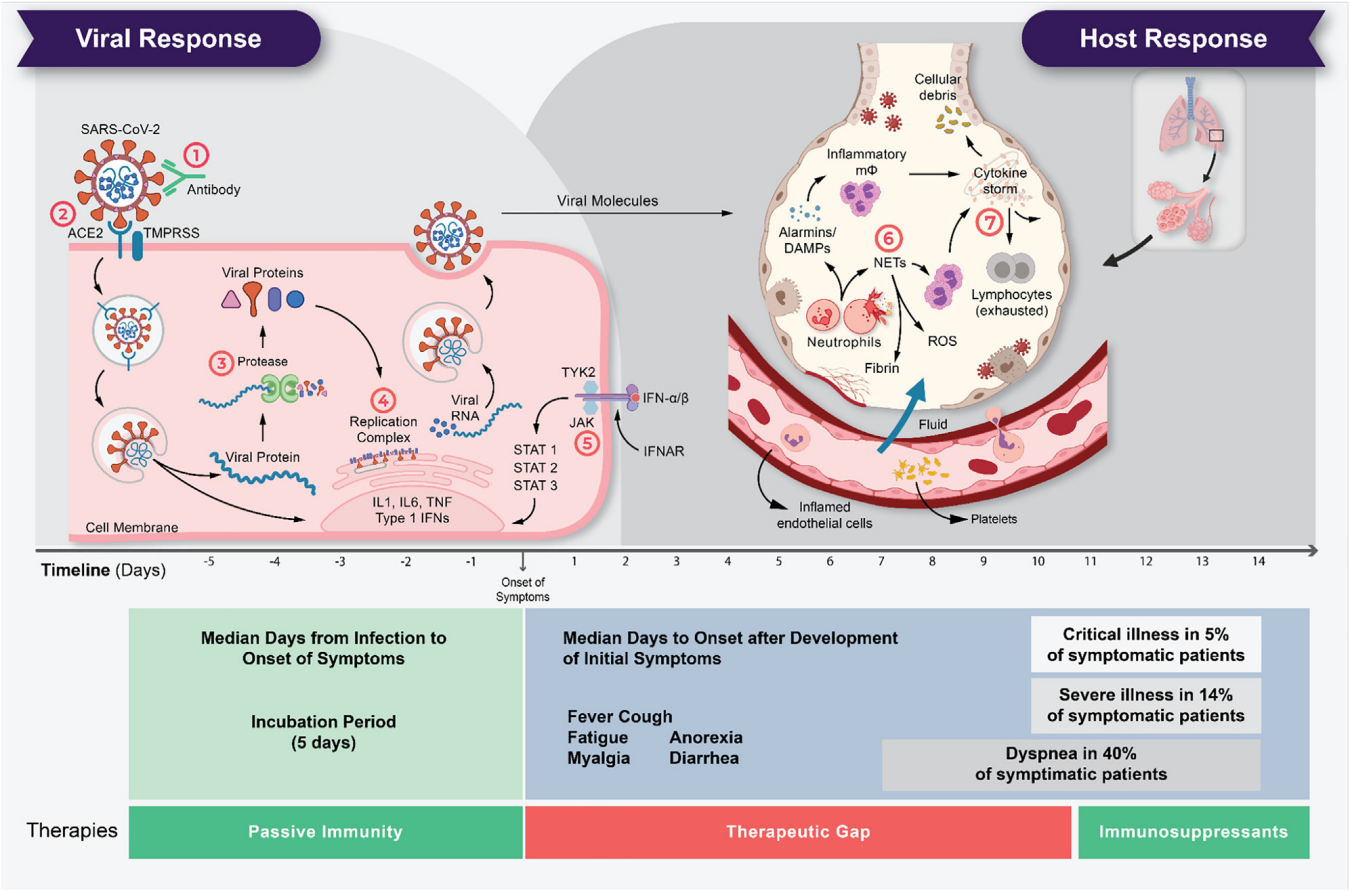
The life cycle of SARS-CoV-2 and the corresponding pathogenesis of COVID-19 display two phases: a viral response and a host-response phase In the viral response phase, the virus enters the host cell and viral replication begins. Approximately five days after infection and successful replication, initial mild and moderate symptoms such as fever, cough, fatigue, anorexia, myalgia, and diarrhea are observed in conjunction with a decrease in lymphocyte cell count (lymphopenia). The following host-response phase determines the severity of the disease: in some patients, uncontrolled overreaction of the immune system—so-called virus-induced immunopathology—requires hospitalization and respiratory support due to ARDS. Thus, severe cases of COVID-19 originate from an immune overreaction rather than from the viral infection itself. Currently, there are seven drug mechanisms described: ① Passive immunity; ② Entry inhibitors; ③ Protease inhibitors; ④ Polymerase inhibitors; ⑤ JAK inhibitors; ⑥ NETosis inhibitors; ⑦ Immunosuppressants.

The role of high neutrophil cell count in critical illness due to COVID-19 can be explained by the previously reported disease mechanism.^3^^,^^27^ Neutrophils are white blood cells and an important component of our host defense against invading pathogens. Critical illness in COVID-19 is characterized by infiltration of the lungs with macrophages and neutrophils that cause diffuse lung alveolar damage, the histological equivalent to ARDS (Figure 4).^26^^,^^28^^,^^29^ Neutrophils release NETs, web-like structures of nucleic acids wrapped with histones that can detain viral particles, through NETosis, a regulated form of neutrophil cell death.^30^ However, ineffective clearance and regulation of NETs result in pathological effects such as thromboinflammation^31^ and endothelial damage.^32^

Our analysis identified two putative drug targets—CDK6 and JAK1/2. We next tested inhibitors of these proteins in a previously published NETosis assay.^32^ We additionally tested colchicine and sabizabulin, which have been proposed to inhibit neutrophil function but whose drug targets were not identified in our GWAS. Indeed, both palbociclib and baricitinib suppressed PMA-induced NETosis. For palbociclib, this had been previously described,^19^ but surprisingly, baricitinib turned out to be a significantly better inhibitor of NETosis. Baricitinib is already approved as a drug against critical illness due to COVID-19,^33^ but the underlying mechanism remains unclear. It has been shown in primate studies that baricitinib inhibits NETosis *in vivo*^34^ and that this appears to be the driving factor in treating critical illness in COVID-19, which is supported by our *ex vivo* data.

We also tested a second NETosis pathway, triggered with calcium ionophore instead of PMA. Here, we observed a suppressive effect with colchicine and sabizabulin. The different behavior due to the used inducer needs further investigations. Unlike palbociclib, baricitinib also had a suppressive effect on calcium ionophore-induced NETs, confirming its clinical utility.

In parallel, we performed MR with neutrophil count as exposure and critically ill COVID-19 course as outcome. The literature describes either no effect^35^ or a slightly negative association^36^ for this scenario. In our experiments, we saw the same result depending on how strictly clumping parameters were selected according to linkage disequilibrium (LD). If clumping was strict, we saw no effect. When we selected more variables due to a less stringent LD threshold, we found that a higher number of neutrophil cells seem to protect against the critical illness in COVID-19. However, the role of neutrophils as a driver of critical illness due to COVID-19 has been clearly described in the literature (see earlier text). This MR result, which is contrary to clinical observation, can be explained by sample size in a manner analogous to the discussion of regression analyses with rs445. As our statistical power analysis has shown, large sample sizes are needed to obtain a large number of gene variants with strong effect sizes. MR only works if a sufficient number of gene variants (instrument variables) with strong effect sizes for exposure and outcome are available. The summary statistics of neutrophil cell count and severe COVID-19 progression underlying MR show an imbalance of sample sizes: the statistics of the neutrophil cell count are based on 408,112 cases, whereas the statistics of critical illness in COVID-19 are based on only 5,582 cases. Ultimately, this leads to insufficient overlap of variables with the necessary effect size to generate a signal in MR. The artificial extension of the overlap by a less strict LD threshold seems to favor the amplification of false signals.

In conclusion, identifying drug targets from GWA data is challenging because of sample sizes limited by patient numbers and the accompanying high dimensionality of the data structure. In addition, GWA studies only reflect associations and do not provide information on causality. In contrast, we have developed a workflow that enables the identification of causal drug targets via the identification and investigation of disease-causing traits. By focusing on the genetics of disease-causing traits, we can leverage larger sample sizes to reveal rare gene variants with small effect sizes. We applied our workflow to critical illness in COVID-19 and could identify high neutrophil count as a possible causal driver of the disease. Based on these findings, we identified CDK6 and JAK inhibitors as potential treatments and successfully tested them in an *ex vivo* NET formation assay. Clinical efficacy against critical illness due to COVID-19 of CDK6 inhibitor palbociclib against severe COVID-19 has been previously reported,^37^ whereas JAK inhibitor baricitinib has even been approved by the Food and Drug Administration (FDA).^33^ The causal inference analysis pipeline developed in this work has been made publically available (github.com/biotxai/covid-19), and a generalized version in the form of a software package called SynTrial is being developed. The package is a complementary addition to MR that can be applied to any number of other indications.

### Limitations of the study

The statistical methods used in this paper come with the standard limitations that include potential biases in retrospective data, risk of false-positive results due to multiple comparisons, limited power to detect small effect sizes, and challenges in generalizing findings due to population-specific variations.

## STAR★Methods

### Key resources table

**Table:** 

REAGENT or RESOURCE	SOURCE	IDENTIFIER
**Chemicals, peptides, and recombinant proteins**		

Baricitinib	Cambridge Bioscience	CAY16707 https://www.bioscience.co.uk
Calcium ionophore A23187	Merck	C7522 https://www.sigmaaldrich.com
Carfilzomib	Cambridge Bioscience	C0271 https://www.bioscience.co.uk
Colchicine	Sigma	C9754
Palbociclib	Sigma	PZ0383
PMA (Phorbol 12-myristate 13-acetate)	Merck	P1585 https://www.sigmaaldrich.com
Sabizabulin	Cambridge Bioscience	HY-120599 https://www.bioscience.co.uk

**Software and algorithms**		

REGENIE	Mbatchou et al.^40^	https://imagej.nih.gov/ij/
R (version 3.6.2)	The R Project for Statistical Computing	https://www.r-project.org/
ieugwasr	Gibran Hemani	https://github.com/MRCIEU/ieugwasr
Syntrial	This paper	https://github.com/biotxai/covid19

**Other**		

UK Biobank project (download October 25, 2021)	UK Biobank	https://www.ukbiobank.ac.uk/
UK Biobank project COVID-19 test results up until 18th October 2021	UK Biobank	https://www.ukbiobank.ac.uk/
Neutrophil Cell Count GWAS (downloaded January 15th 2021)	Vuckovic et al.^23^	https://www.ebi.ac.uk/gwas/studies/GCST90002398
GWAS meta-analyses round 5 release date January 18th 2021	COVID19hg	https://www.covid19hg.org/results

### Resource availability

#### Lead contact

Further information and requests for resources and reagents should be directed to and will be fulfilled by the lead contact, Marco Schmidt (ms@biotx.ai).

#### Materials availability

This study did not generate new unique reagents.

### Experimental model and study participant details

Our study was approved by the UK NHS Research Ethics Committee and blood donations from healthy volunteers were collected after obtaining informed consent. The study includes samples from 3 male and 3 female donors, all of European ethnicity and within the age range of 22 and 41 years. There were no statistically significant differences in responses between males and females.

### Method details

#### Recruitment of cases and controls

We downloaded the rich information made available by the UK Biobank project on October 25, 2021 and COVID-19 test results up until 18^th^ October 2021 were collected. We created four cohorts for which inclusion and exclusion criteria are outlined below.

First, COVID-19 cases were defined as reported previously.^8^ Briefly, 1,505 severe cases were defined as patients who died or were hospitalized due to COVID-19 (cause of death or diagnosis containing ICD10 codes U07.1 or U07.2) or were ventilated (operation codes E85.∗) in 2020 or 2021 and tested positive for SARS-CoV-2 infection. Second, Individuals that were tested positive for SARS-CoV-2, but did not die or were critical due to COVID-19 and were not ventilated, were defined as potential mild COVID-19 controls. Third, the infectious disease phenotype was created based on UK Biobank data for respiratory infections, ARDS, influenza, and pneumonia with hospitalization or death as a result. We aggregated hospital in-patient and death register data for ICD codes corresponding to J00-J06 (“Acute upper respiratory infections”), J09-J18 (“Influenza and pneumonia”), J20-J22 (“Other acute lower respiratory infections”), and J80 (ARDS), yielding 42,065 cases. Finally, the remaining individuals from the UK Biobank were defined as potential healthy controls.

For both cohorts, cases and controls were filtered for European ancestry (“White”, “British”, “Irish”, and “Any other white background”) since they represented the largest portion of the available cases (94.4%). This helped to isolate ethnicity-dependent discoveries, at the cost of reducing the scope of our study. Finally, individuals with missing age and sex information were discarded. For both cohorts, controls were then matched to the same number of cases based on age and sex.

Variants reported by Pairo-Castineira et al.^8^ and Ellinghaus et al.^7^ as well as variants reported by the ClinVar database^38^ for the genes reported by the papers were included in the dataset.

#### Screening for significant traits

The UK Biobank contains data on biological samples taken upon registration of individuals to the program, years before potential infection. Measurements include 33 blood cell counts and 30 blood biochemistry markers, and body mass index. In order to identify traits that are significantly different between the infectious disease cohort and healthy controls, we performed either independent two-sample t-test or Wilcox rank-sum test from the R package stats (www.rdocumentation.org/packages/stats/versions/3.6.2), depending on whether the trait follows a normal distribution or not. We applied a Bonferroni-corrected p value threshold of *p* < α/n = 0.05/64. In five instances, the p values were too small to be represented properly, and were instead set to 1.0E-297.

#### Regression modeling

Logistic regression models were fitted using the *glm* function in R (www.R-project.org).

#### Collinearity testing

We estimated Pearson correlation coefficients between traits and applied a collinearity threshold of 0.5 and subset from the data the trait pairs where the absolute collinearity estimate is greater or equal to the collinearity threshold. We then iteratively removed the trait with the lower regression coefficient of that pair.

#### Drop-one analysis

A drop-one model comparison procedure was performed using the *drop1()* function in R (www.R-project.org) in order to determine whether each of a set of traits accounts for unique variance in critically ill COVID-19 disease status. The formula of BMI + high light scatter reticulocyte count + erythrocyte distribution width + neutrophil count + lymphocyte count + alkaline phosphatase + apolipoprotein A + C-reactive protein + cystatin C + gamma glutamyltransferase + glucose + SHBG + triglycerides + vitamin D was used to predict critical illness due to COVID-19. Single terms were deleted and the F value is calculated to perform an F-test to derive the Pr(>F) value, where low values indicate that a model that does not include this term is significantly different from the full model.

#### Propensity score analysis

Using the method of Imai and Van Dyk,^14^ individuals are split into deciles who have a similar propensity for a treatment (neutrophil count) given the covariates (the risk factors age, sex, BMI, C-reactive protein, cystatin C (as a proxy for cardiovascular disease), alanine aminotransferase (as a proxy for chronic liver disease), and creatinine (as a proxy for chronic kidney disease)). We then estimated the effect of treatment on severe COVID-19 within each of the groups. The effect across these groups is examined and the average effect of treatment is calculated over the groups to give an estimate of effect of treatment independent of the covariates.

#### GWA analysis

Prior to genome-wide association analysis, we took steps to remove biases by submitting UK Biobank genotypes to a series of quality control steps using PLINK 2.0.^39^ First, we extracted variants on autosomal chromosomes. Then we filtered samples for European ancestry and further dropped all samples with missing data for the phenotype of interest (neutrophil cell count) or for any of the following covariates: age, sex, BMI, and genetic principal components. These initial filtering steps left us with 444,114 samples and 784,256 variants. Next, we filtered variants for minor allele frequency (MAF) using a threshold of 0.01 for the aggregate frequency and count of non-major alleles, since extremely rare alleles may indicate genotyping errors and furthermore are cases where power for detecting variant-to-phenotype associations is lacking. We then filtered variants based on missingness in the dataset with a threshold of 0.1, excluding variants where genotyping information is unavailable or of poor quality for more than 10 percent of subjects. Next, as an additional guard against genotyping errors, variants deviating from Hardy-Weinberg equilibrium were removed where exact test p values fell below the threshold of 1E-15. We then filtered samples with a missingness threshold of 0.1, excluding samples where genotyping information is unavailable or of poor quality for more than 10 percent of variants. This yielded a final dataset with a total of 444,109 samples and of 509,485 variants. Finally, a genome-wide association analysis was performed in two steps with REGENIE.^40^ In the first step, a whole genome regression model was fitted using ridge regression, and in the second step, variants were tested for association with the continuous neutrophil cell count phenotype conditioned on the prediction of the model from the prior step using the “leave one chromosome out” scheme (LOCO) to avoid proximal contamination. In both steps, the first four genetic principal components were included as covariates.

#### Neutrophil isolation and NET assay

Blood was collected into EDTA tubes (BD Vacutainer), and neutrophils were isolated by negative immunomagnetic selection using EasySep, (STEMCELL Technologies), as per the manufacturer’s instructions.

NETs were quantified using SYTOX Green REF PMID: 31628160 (1 μM, Invitrogen), which was added to RPMI-1640 w/o phenol red (Gibco). Freshly isolated neutrophils were resuspended in this medium, containing the indicated concentrations of chemical inhibitors (baricitinib, carfilzomib, colchicine, palbociclib, and sabizabulin) or vehicle control (DMSO) and seeded in 96-well flat-bottomed plates, at a density of 3 × 10^4^ cells per well. Cells were pre-incubated with the inhibitors for 40 min at 37°C, before stimulation with 50 nM phorbol 12-myristate 13-acetate (PMA) or 2.5 μM A23187. Immediately after stimulation the plate was read on an Incucyte Zoom (Essen BioScience), with further reads every 20 min for 4 h. Induction of NETs was quantified by analyzing the total green area for each timepoint.

#### Mendelian randomization

We used independent GWAS summary data for neutrophil cell count (exposure) published by Vuckovic et al.^23^ (GCST90002398 downloaded January 15th 2021) and summary data for critically ill COVID-19 status (outcome) published by the COVID-19 Host Genetics Initiative (https://www.covid19hg.org/results - COVID19hg GWAS meta-analyses round 5 release date January 18th 2021). Two-sample MR analyses were done as previously described^10^ and implemented in the R package ieugwasr. Briefly, MR aims to estimate the causal effect between an exposure (e.g., neutrophil cell count) and an outcome (e.g., severe COVID-19 disease progression) based on a number of instrumental variables (e.g., SNPs). Selection of instrumental variables is an important step and reducing redundant information across them is essential. In the case of SNPs, linkage disequilibrium can be used to extract only variants with independent contributions in a process called clumping. Different stringencies are applied to clumping via a squared correlation threshold (r^2^) at which value variants are considered to carry redundant information. A more lenient parameter includes more variants. Since an MR analysis requires an overlap of instrumental variables across exposure and outcome datasets, tweaking the clumping parameters modulates the SNP overlap to enable an MR analysis.

### Quantification and statistical analysis

The calculation of the effect size required to achieve a certain statistical power based on a fixed p value threshold is based on https://www.mv.helsinki.fi/home/mjxpirin/GWAS_course/material/GWAS3.html. Due to the high computational cost, a random slice of 10% of the variants from the GWAS was used in these calculations.

All statistical analyses were performed in R version 3.6.2.

## Data Availability

• All data reported in this paper will be shared by the lead contact upon request. DOIs are listed in the key resources table. • Original codes are available on request to the corresponding authors and are also publicly available at https://github.com/biotxai/covid19. • Any additional information required to reanalyze the data reported in this paper is available from the lead contact upon request.
