# Supplementary material for: Exploring disease-causing traits for drug repurposing in critically ill COVID-19 patients: A causal inference approach
Source: iScience. 2023 Oct 12;26(11):108185. doi: 10.1016/j.isci.2023.108185 (PMC10641251; doi:10.1016/j.isci.2023.108185)
Supplement: Document S1. Figures S1–S9 and Tables S1–S6 [file mmc1.pdf]

iScience, Volume *26*

Supplemental information

Exploring disease-causing traits for drug repurposing in critically ill COVID-19 patients: A causal inference approach

Hannes A. Baukmann, Justin L. Cope, Colin Bannard, Alexander R.E.C. Schwinges, Margaretha R.J. Lamparter, Sarah Groves, Charles N.J. Ravarani, Borko Amulic, Joern E. Klinger, and Marco F. Schmidt

Supplementary information

SI Figure 1

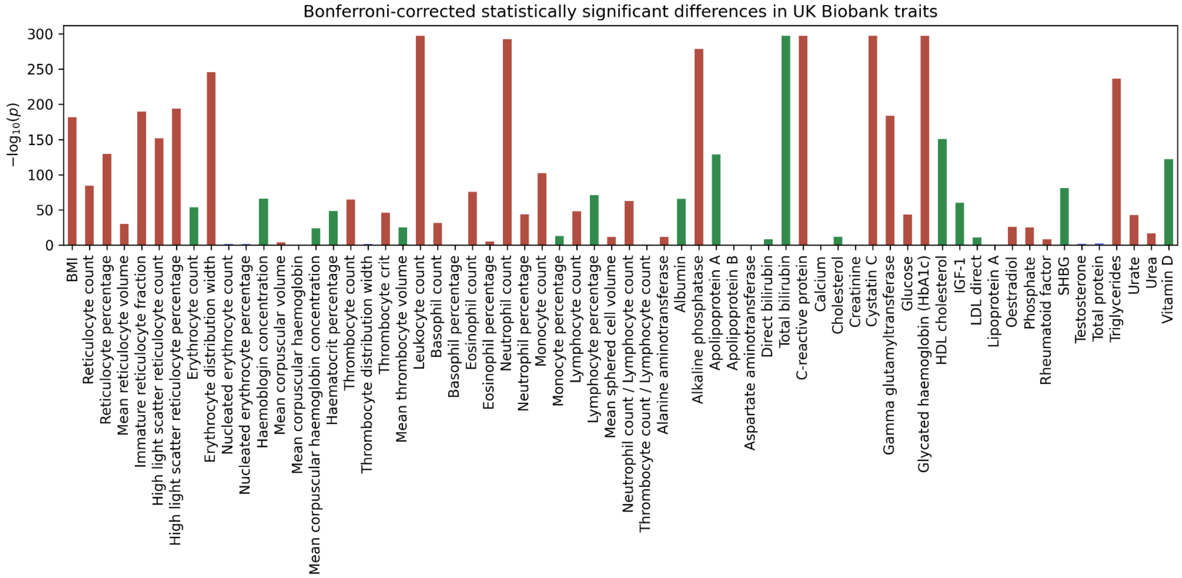

SI Fig. 1. Screening for UKB traits associated with infectious disease, related STAR to Methods. Bonferroni-corrected statistically significant differences in 64 traits identified using independent two-sample *t*-test and Mann-Whitney U test. Trait measurement distributions of infectious disease cases and healthy controls were compared and the p-values, measuring the likelihood of differences by random chance, are reported with the negative logarithm base 10 (see Methods - Screening for significant traits). Red and green columns indicate traits that are significantly increased in infectious disease cases or healthy controls, respectively.

SI Figure 2

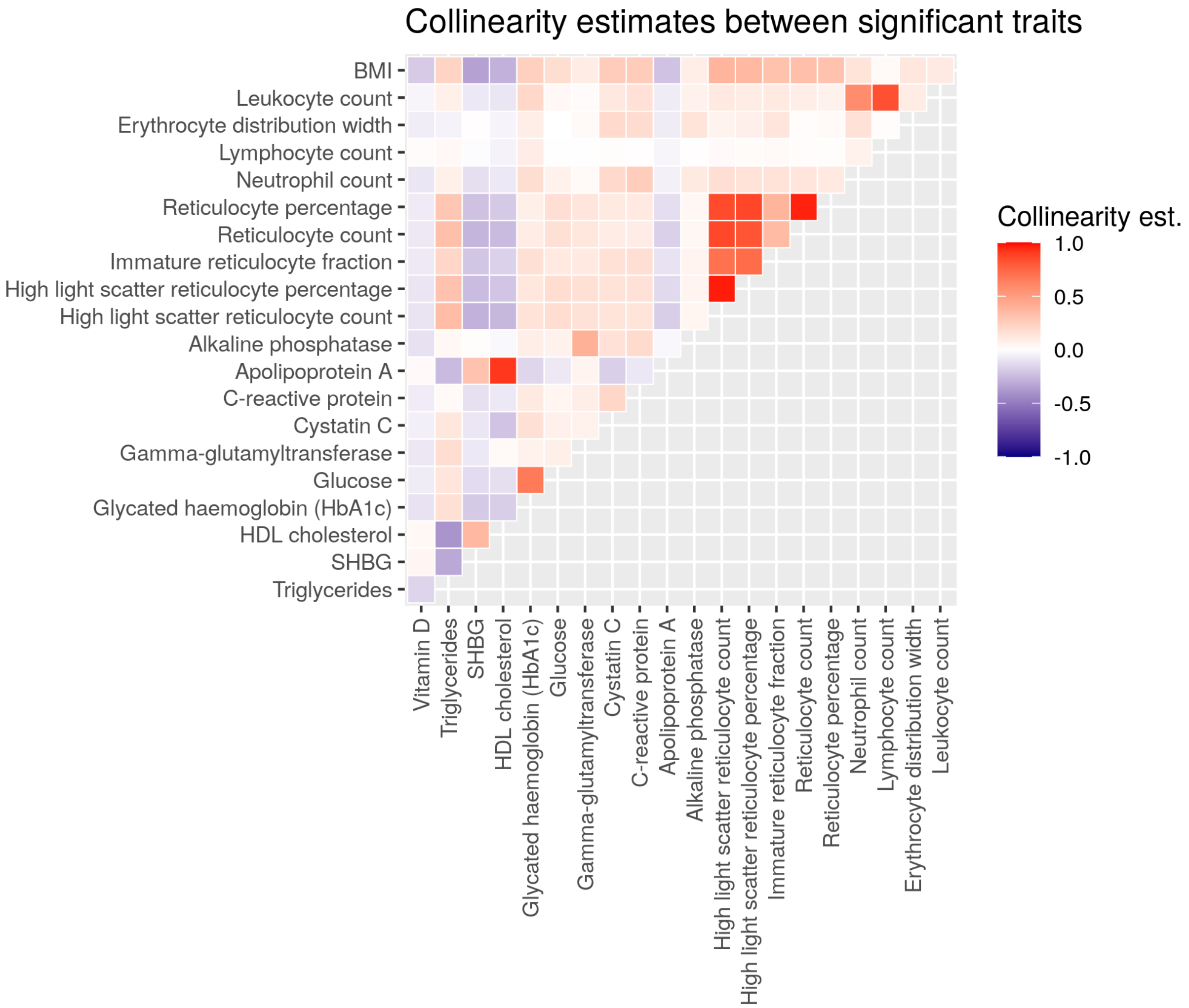

SI Fig. 2. Collinearity estimates between significant traits, related to STAR Methods. Heatmap showing collinearity estimates between the 21 traits significant in regression analysis. Highly collinear traits (absolute collinearity greater than 0.5) are removed as keeping both would include redundant information in downstream analysis.

SI Figure 3

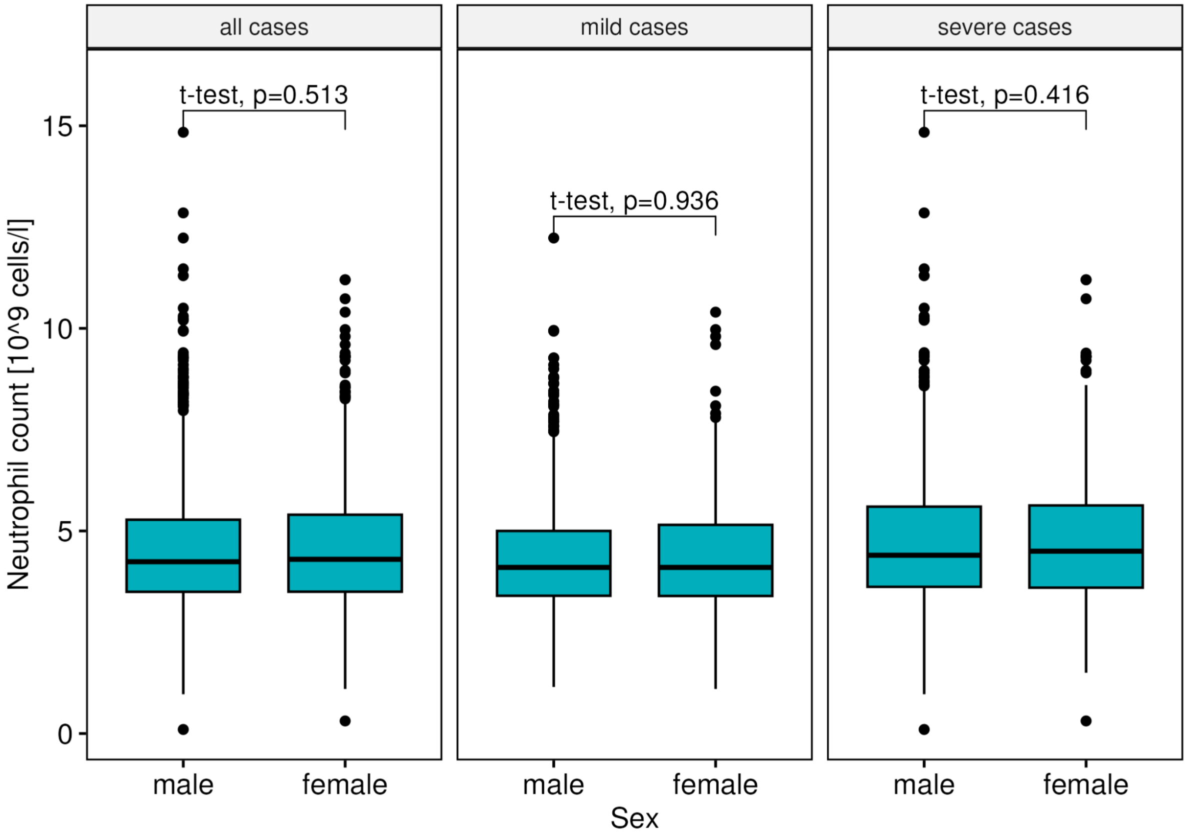

SI Fig. 3. Neutrophil cell count is independent of sex across cases and controls, related to STAR Methods. T-tests were used to assess differences in neutrophil counts between males and females. No significant differences were observed when considering all cases, or only mild cases or severe cases.

SI Figure 4

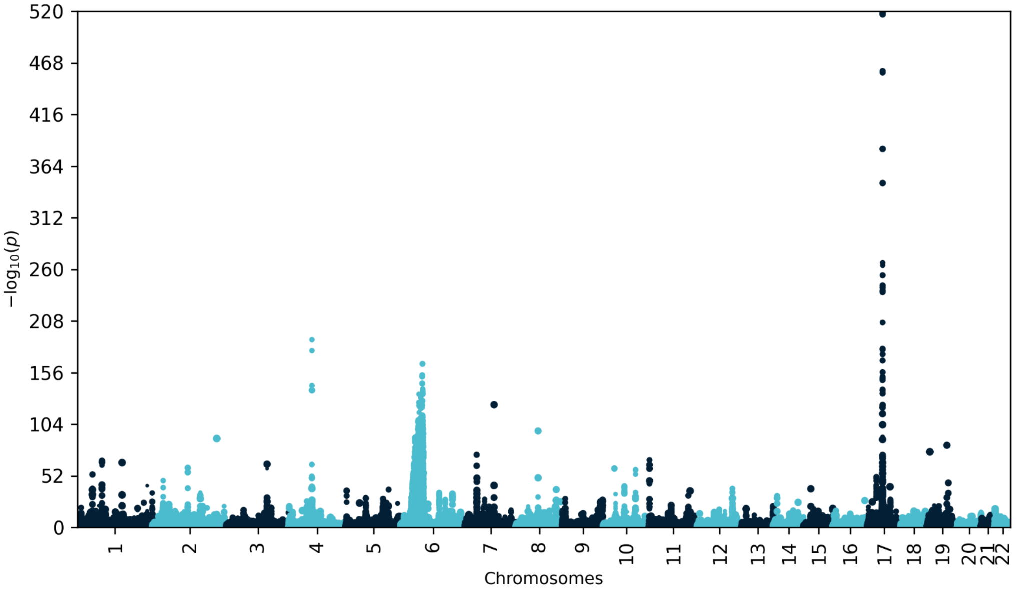

SI Fig. 4. Genetic loci associated with neutrophil cell count, related STAR to Methods. Manhattan plot of UKB participants with measured neutrophil cell counts available (n = 444,109). A number of genomic loci show an association with neutrophil cell count, with chromosome 17 harboring a particularly significant locus. This highlights the increased statistical power of a GWAS when using patients' measured traits, which provides a larger pool of subjects, as opposed to disease status, which is often limiting.

SI Figure 5

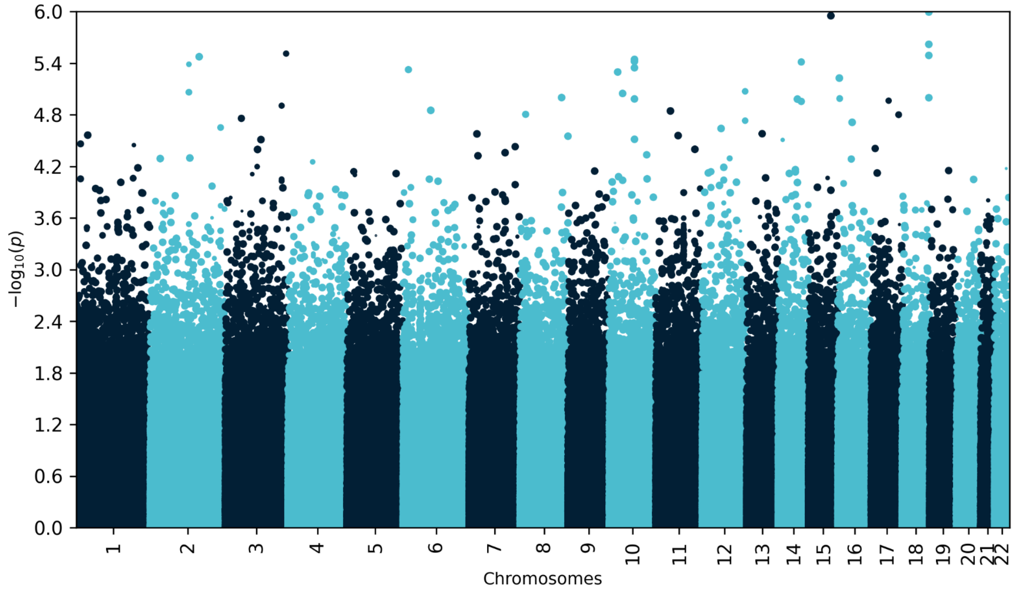

SI Fig. 5. Manhattan plot of neutrophil cell count using only COVID-19 cases and a matching number of controls, related STAR to Methods. With the limited number of subjects available (n = 3,010), the statistically significant genomic loci associated with neutrophil cell count are not identified due to lack of power.

SI Figure 6

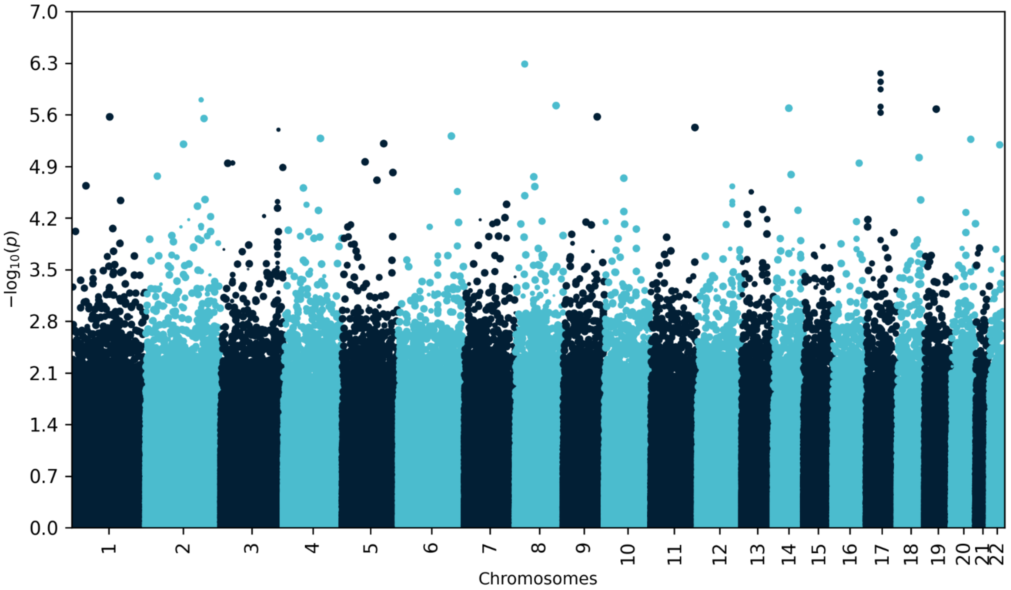

SI Fig. 6. Manhattan plot of neutrophil cell count using a random subset of the filtered population, related STAR to Methods. The random subset (n = 3,010) creates a control sample of the same size as the COVID-19 cohort used in this study, accounting for any of its particular makeup, while highlighting the limited power of smaller sample sizes.

SI Figure 7

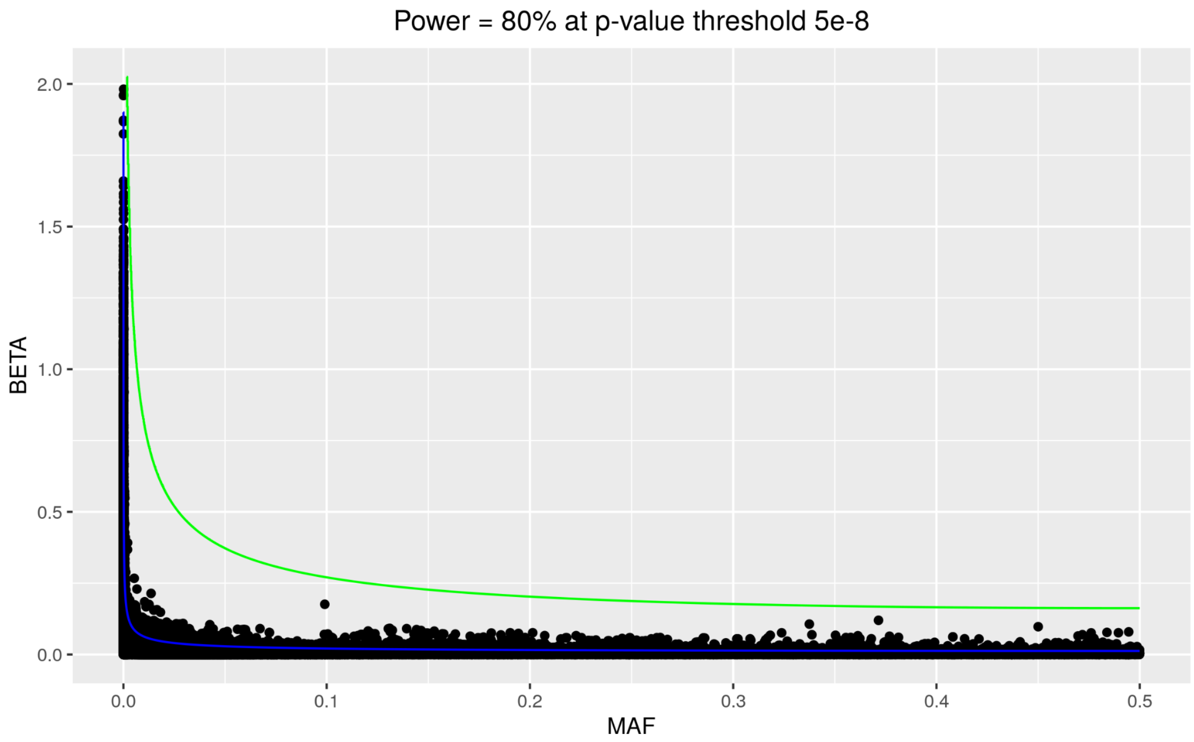

SI Fig. 7. Statistical power calculations, related STAR to Methods. Dots represent variants and their effect size (beta, log Odds Ratio) for neutrophil count as determined by the GWAS (n = 444,109). The lines represent the effect size required to achieve a statistical power of 80% at a *p*-value threshold of 1E-15 in the full GWAS (blue) and the GWAS with a random subset of the same size and the cases and controls for severe COVID-19 (green) (n = 3,010).

SI Figure 8

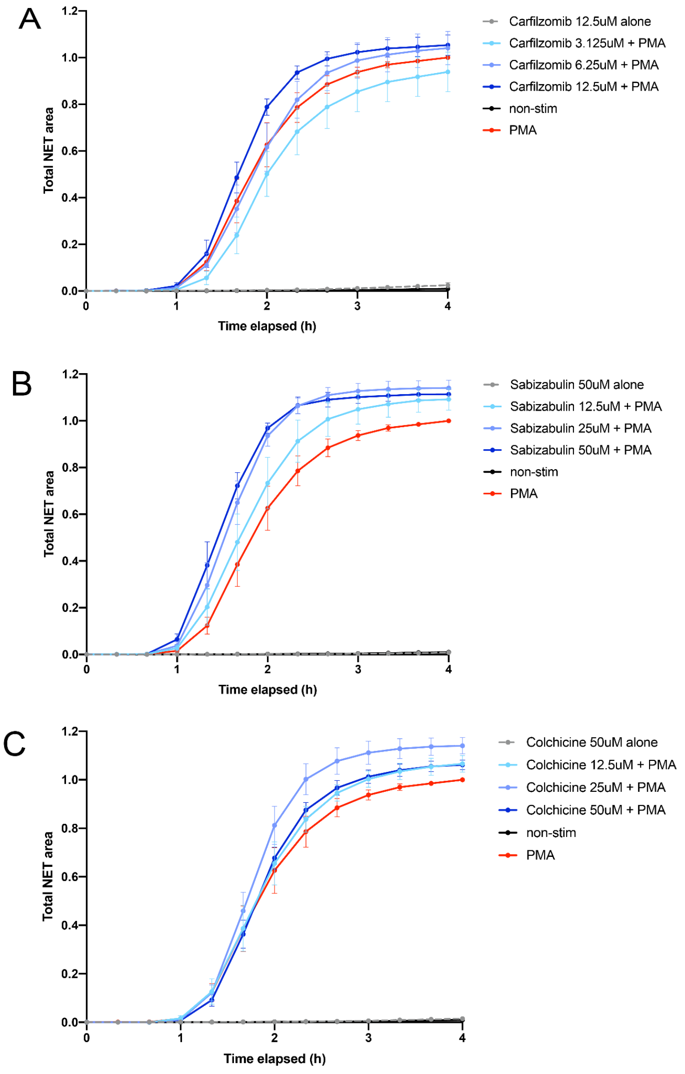

SI Fig. 8. Effects of drugs on NET formation inhibition, related STAR to Methods. Carfilzomib, sabizabulin and colchicine fail to inhibit PMA-induced NETosis. Neutrophils from healthy donors (n = 6) were incubated with carfilzomib (A) or sabizabulin (B) or colchicine (C) for 45 minutes at the indicated concentrations, before addition of PMA (50 nM). Total NET area was quantified using live cell imaging over 4h in the presence of SYTOX green.

SI Figure 9

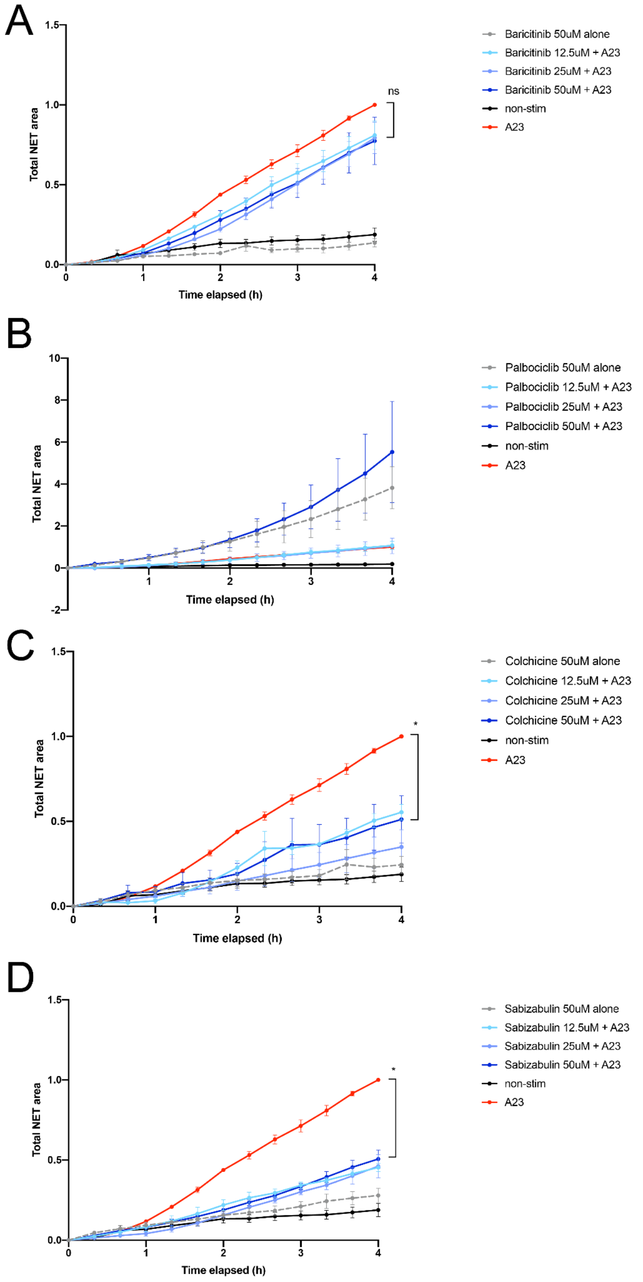

SI Fig. 9. Effect of inhibitors on NETs triggered by A23187, related STAR to Methods. Neutrophils from healthy donors (n = 3) were incubated with baricitinib (A), palbociclib (B), colchicine (C) or sabuzabulin (D) for 45 minutes at the indicated concentrations, before addition of A23187 (2.5 μM). Total NET area was quantified using live cell imaging over 4h in the presence of SYTOX green.

SI Table 1

SI Tab. 1. Critical illness in COVID-19 regressed on the traits significantly different between infectious disease cases and healthy controls, related to STAR Methods. Significance thresholds are indicated by asterisks, where three asterisks indicate *p*-values below 0.001/51, two indicate *p*-values below 0.01/51, and one asterisk indicates *p*-values below 0.05/51.

Trait	Estimate	SE	*p*-value
**BMI**	0.04719	0.007028	1.89163E-11 ***
**Reticulocyte count**	7.60919	1.496925	3.71103E-07 ***
**Reticulocyte percentage**	0.37954	0.071004	9.02311E-08 ***
**Mean reticulocyte volume**	0.00414	0.004666	3.74834E-01
**Immature reticulocyte fraction**	3.64822	0.610483	2.28739E-09 ***
**High light scatter reticulocyte count**	22.60823	3.765371	1.92216E-09 ***
**High light scatter reticulocyte percentage**	1.06815	0.173818	7.98365E-10 ***
**Erythrocyte count**	-0.01703	0.086263	8.43490E-01
**Erythrocyte distribution width**	0.16615	0.039526	2.62767E-05 **
**Haemoblogin concentration**	-0.00891	0.029668	7.63919E-01
**Mean corpuscular volume**	-0.00217	0.008046	7.87806E-01
**Mean corpuscular haemoglobin concentration**	0.02570	0.035277	4.66262E-01
**Haematocit percentage**	-0.00465	0.010271	6.50538E-01
**Thrombocyte count**	0.00168	0.000604	5.24684E-03
**Thrombocyte crit**	1.72371	0.730985	1.83706E-02
**Mean thrombocyte volume**	-0.04106	0.034446	2.33295E-01
**Leukocyte count**	0.14942	0.019929	6.49568E-14 ***
**Basophil count**	2.35277	0.795494	3.10016E-03
**Eosinophil count**	0.23842	0.266235	3.70503E-01
**Eosinophil percentage**	-0.03716	0.020737	7.31587E-02
**Neutrophil count**	0.170778	0.025309	1.50164E-11 ***
**Neutrophil percentage**	0.00943	0.004216	2.52423E-02
**Monocyte count**	0.33506	0.155611	3.13015E-02
**Monocyte percentage**	-0.01740	0.012125	1.51270E-01
**Lymphocyte count**	0.23706	0.055760	2.12451E-05 **

**Lymphocyte percentage**	-0.00802	0.004870	9.96131E-02
**Mean sphered cell volume**	0.00048	0.006796	9.44011E-01
**Neutrophil count / Lymphocyte count**	0.08736	0.028739	2.36929E-03
**Alanine aminotransferase**	0.00637	0.001440	9.61590E-06 ***
**Albumin**	-0.00503	0.013993	7.19078E-01
**Alkaline phosphatase**	0.00507	0.002474	4.03809E-02
**Apolipoprotein A**	-0.53989	0.145500	2.06757E-04 *
**Direct bilirubin**	0.00907	0.044307	8.37844E-01
**Total bilirubin**	-0.02864	0.009055	1.56234E-03
**C-reactive protein**	0.03173	0.008310	1.34563E-04 **
**Cholesterol**	-0.01326	0.031792	6.76677E-01
**Cystatin C**	1.09557	0.195222	2.00061E-08 ***
**Gamma glutamyltransferase**	0.00315	0.000801	8.30303E-05 **
**Glucose**	0.13716	0.026707	2.80836E-07 ***
**Glycated haemoglobin (HbA1c)**	0.03611	0.005021	6.38047E-13 ***
**HDL cholesterol**	-0.47517	0.106974	8.91739E-06 ***
**IGF-1**	-0.01185	0.006495	6.81625E-02
**LDL direct**	-0.01949	0.042154	6.43797E-01
**Oestradiol**	-0.00038	0.000409	3.57671E-01
**Phosphate**	0.01507	0.231906	9.48200E-01
**Rheumatoid factor**	0.00997	0.003762	8.03311E-03
**SHBG**	-0.00587	0.001616	2.83598E-04 *
**Triglycerides**	0.24894	0.037066	1.86505E-11 ***
**Urate**	0.00142	0.000451	1.60977E-03
**Urea**	0.04115	0.022342	6.54756E-02
**Vitamin D**	-0.00998	0.001772	1.79162E-08 ***

SI Table 2

SI Tab. 2. Collinearity of traits with significant regressions, related to STAR Methods. Collinearity estimates greater than 0.5 between the 21 traits significant in regression analysis. Highly collinear traits are removed as keeping both would include redundant information in downstream analysis. Therefore one trait within a highly collinear pair was filtered out, giving preference to the trait with the higher regression coefficient with COVID-19 status.

Trait 1	Trait 2	Collinearity estimate	Regression estimate trait 1	Regression estimate trait 2	Trait removed
High light scatter reticulocyte percentage	High light scatter reticulocyte count	0.9738	1.0682	22.6082	Trait 1
Reticulocyte percentage	Reticulocyte count	0.9639	0.3795	7.6092	Trait 1
Apolipoprotein A	HDL cholesterol	0.9181	-0.5399	-0.4752	Trait 2
Reticulocyte percentage	High light scatter reticulocyte percentage	0.8743	0.3795	1.0682	both
Reticulocyte count	High light scatter reticulocyte count	0.8694	7.6092	22.6082	Trait 1
Reticulocyte percentage	High light scatter reticulocyte count	0.8604	0.3795	22.6082	Trait 1
Leukocyte count	Lymphocyte count	0.8391	0.1494	0.2371	Trait 1
Reticulocyte count	High light scatter reticulocyte percentage	0.8244	7.6092	1.0682	both
Immature reticulocyte fraction	High light scatter reticulocyte percentage	0.7328	3.6482	1.0682	Trait 2
Immature reticulocyte fraction	High light scatter reticulocyte count	0.7106	3.6482	22.6082	Trait 1
Glucose	Glycated haemoglobin (HbA1c)	0.6706	0.1372	0.0361	Trait 2
Leukocyte count	Neutrophil count	0.5886	0.1494	0.1708	Trait 1

SI Table 3

SI Tab. 3. Drop-one analysis of traits filtered by collinearity analysis, related to STAR Methods. F values and their probabilities Pr(>F) values of traits determined in drop-one analysis. Significance thresholds are indicated by asterisks, where three asterisks indicate *p*-values below 0.001/14, two indicate p-values below 0.01/14, and one asterisk indicates *p*-values below 0.05/14.

Trait	F value	Pr(>F)
**BMI**	0.741657	0.3892078
**High light scatter reticulocyte count**	1.761507	0.1845500
**Erythrocyte distribution width**	1.984917	0.1589896
**Neutrophil count**	9.562278	0.0020067 *
**Lymphocyte count**	7.840082	0.0051467
**Alkaline phosphatase**	1.275999	0.2587456
**Apolipoprotein A**	2.111558	0.1463078
**C-reactive protein**	0.061001	0.8049391
**Cystatin C**	3.574881	0.0587677
**Gamma glutamyltransferase**	2.696038	0.1007155
**Glucose**	7.641474	0.0057432
**SHBG**	0.015972	0.8994413
**triglycerides**	4.532300	0.0333520
**Vitamin D**	8.091378	0.0044815

SI Table 4

SI Tab. 4. Neutrophil cell count propensity scores across cases and controls, related to STAR Methods. Neutrophil cell count [10^9 cells / liter] across cases and controls in the propensity score deciles (see Methods - Propensity Score Analysis) obtained by splitting individuals into ten groups with a similar propensity for a treatment (neutrophil cell count) controlling for the covariates (age, sex, BMI, C-reactive protein, cystatin C, alanine aminotransferase, and creatinine). The estimates in the table summarize the average neutrophil cell count for each group for both cases and controls.

**Decile number**	**1st**	**2nd**	**3rd**	**4th**	**5th**	**6th**	**7th**	**8th**	**9th**	**10th**
**ctrls**	3.70	3.83	4.18	3.98	4.29	4.48	4.50	4.79	4.88	5.23
**cases**	3.98	4.20	4.26	4.57	4.60	4.53	4.70	4.85	4.92	5.55

SI Table 5

SI Tab. 5. Drugs and drug targets with variants significantly associated with neutrophil cell count, related to STAR Methods. Genes and FDA-approved drugs for variants with -log *p*-values greater than 10 for neutrophil cell count. RS ID stands for Reference SNP cluster ID and provides unique standard identifiers for SNPs.

RS ID	Chrom:Pos	-log *p*	Gene	Drug	Chembl ID
**rs57968500**	17:38145828	516.737	*PSMD3*	Bortezomib	CHEMBL325041
	17:38145828	516.737	*PSMD3*	Carfilzomib	CHEMBL451887
**rs56030650**	17:38131187	459.752	*GSDMA*	NA	NA
**rs3859191**	17:38128714	458.236	*GSDMA*	NA	NA
**rs8077456**	17:38128765	381.308	*GSDMA*	NA	NA
**rs34003767**	17:38194296	346.912	*MED24*	NA	NA
**rs3902025**	17:38119254	254.000	*GSDMA*	NA	NA
**rs3894194**	17:38121993	243.737	*GSDMA*	NA	NA
**rs4795406**	17:38100134	238.452	*LRRC3C*	NA	NA
**rs4795405**	17:38088417	206.491	*LRRC3C*	NA	NA
**rs4795399**	17:38061439	179.991	*GSDMB*	NA	NA
**rs11078928**	17:38064469	179.218	*GSDMB*	NA	NA
**rs7216389**	17:38069949	156.388	*GSDMB*	NA	NA
**rs2290400**	17:38066240	151.803	*GSDMB*	NA	NA
**rs2305479**	17:38062217	149.614	*GSDMB*	NA	NA
**rs907092**	17:37922259	148.403	*IKZF3*	NA	NA
**rs60069701**	4:75044689	138.183	*MTHFD2L*	NA	NA
**rs870829**	17:38068382	136.478	*GSDMB*	NA	NA
**rs9303277**	17:37976469	134.933	*IKZF3*	NA	NA
**rs921650**	17:38069076	134.841	*GSDMB*	NA	NA
**rs445**	7:92408370	123.637	*CDK6*	Palbociclib	CHEMBL189963
	7:92408370	123.637	*CDK6*	Ribociclib	CHEMBL3545110
	7:92408370	123.637	*CDK6*	Fulvestrant	CHEMBL1358
	7:92408370	123.637	*CDK6*	Abemaciclib	CHEMBL3301610
	7:92408370	123.637	*CDK6*	Trilaciclib	CHEMBL3894860

	7:92408370	123.637	*CDK6*	Apremilast	CHEMBL514800
	7:92408370	123.637	*CDK6*	Dexamethasone	CHEMBL384467
**rs141144358**	17:38251385	123.212	*NR1D1*	Lithium	CHEMBL2146126
**rs2102928**	17:38253228	115.612	*NR1D1*	Lithium	CHEMBL2146126
**rs9635726**	17:38020141	114.486	*IKZF3*	NA	NA
**rs4247366**	17:38179374	103.477	*MED24*	NA	NA
**rs11775560**	8:61660163	97.198	*CHD7*	NA	NA
**rs939348**	17:38231853	91.093	*THRA*	Levothyroxine	CHEMBL16244
	17:38231853	91.093	*THRA*	Liothyronine	CHEMBL1544
	17:38231853	91.093	*THRA*	Aspirin	CHEMBL25
	17:38231853	91.093	*THRA*	Lithium	CHEMBL2146126
**rs55799208**	2:218999982	89.572	*CXCR2*	Clotrimazole	CHEMBL104
	2:218999982	89.572	*CXCR2*	Acetylcysteine	CHEMBL600
	2:218999982	89.572	*CXCR2*	Ibuprofen	CHEMBL521
**rs4760**	19:44153100	82.772	*PLAUR*	Filgrastim	CHEMBL1201567
	19:44153100	82.772	*PLAUR*	Ruxolitinib	CHEMBL1789941
**rs1887428**	9:4984530	11.004	*JAK2*	Baricitinib	CHEMBL2105759
	9:4984530	11.004	*JAK2*	Ruxolitinib	CHEMBL1789941

SI Table 6

SI Tab. 6. The Two Sample MR analyzes with varying clumping parameters, related to STAR Methods. With neutrophil cell count as exposure and critically ill COVID-19 status as outcome, results were dependent on the clumping parameters applied, i.e. only significant with lenient clumping parameters The clumping parameter (r^2) gives the squared correlation threshold for clumping, n SNPs is the number of SNPs resulting from the clumping procedure, effect estimate (± standard error) and the p-value result from the MR experiment using the IVW method, and the negative pleiotropy test indicates that results do not show pleiotropy. The asterisk signifies statistical significance with P ≤ 0.05.

**Clumping parameter**	**n SNPs**	**Effect Estimate**	**IVW *p*-value**	**Pleiotropy test**
lenient (r^2 = 0.2)	1,581	–0.1114 ± 0.04433	0.01199 *	negative
strict (r^2 = 0.01)	567	0.0114 ± 0.06987	0.87095	negative
